# Supplementary material for: Design of Hierarchically Tailored Hybrids Based on Nickle Nanocrystal-Decorated Manganese Dioxides for Enhanced Fire Safety of Epoxy Resin
Source: Int J Mol Sci. 2022 Nov 8;23(22):13711. doi: 10.3390/ijms232213711 (PMC9697679; doi:10.3390/ijms232213711)
Supplement: Supplementary file 1 [file ijms-23-13711-s001.zip › ijms-2015762-supplementary.pdf]

International Journal of *Molecular Sciences*

MDPI

Supporting information for

Design of Hierarchically Tailored Hybrids based on Nickle Nanocrystal Decorated Manganese Dioxides for Enhanced Fire Safety of Epoxy Resin

Yao Yuan [1,*], Chen Liang [1], Anthony Chun Yin Yuen [2], Lulu Xu [3], Bin Yu [4], Chengfei Cao [5], and Wei Wang [2,*]

[1] Fujian Provincial Key Laboratory of Functional Materials and Applications, School of Materials Science and Engineering, Xiamen University of Technology, Xiamen 361024, PR China
[2] School of Mechanical and Manufacturing Engineering, University of New South Wales, Sydney, NSW 2052, Australia
[3] School of Materials Science and Engineering, Nanyang Technological University, 50 Nanyang Avenue, Singapore 639798, Singapore
[4] State Key Laboratory of Fire Science, University of Science and Technology of China, Anhui 230026, PR China
[5] Centre for Future Materials, University of Southern Queensland, Springfield Central, 4300, Australia

* Corresponding authors: Dr. Yao Yuan and Dr. Wei Wang
Fax/Tel: + 86-059-26291325
Correspondence: yuanyao@xmut.edu.cn (Yao Yuan) and wei.wang15@unsw.edu.au (Wei Wang)

Additional Supporting Data

Table captions

Table S1 TGA results of pristine EP, EP/MnO_2 and EP/MnO_2@CHS@SA@Ni composites under nitrogen atmosphere.

Figure captions

Figure S1 The synthesis process of MnO_2@Chitosan@SA@Ni hybrids.

Figure S2 XPS survey spectra of MnO_2 and MnO_2@CHS@SA@Ni hybrids.

Figure S3 Mn 2p spectra of MnO_2 and MnO_2@CHS@SA@Ni hybrids.

Figure S4 (a) FTIR spectra and (b) TGA **curves of untreated** MnO_2 and MnO_2@CHS@SA@Ni hybrids.

Figure S5 The enlarged TGA patterns of pristine EP, EP/MnO_2 and EP/MnO_2@CHS@SA@Ni composites under nitrogen.

Figure S6 (a) FPI and (b) FIGRA values of **pristine** EP, EP/MnO_2 and EP/ MnO_2@CHS@SA@Ni composites.

Figure S7 Interior and exterior char residue analysis by using XPS technique. (a) XPS survey spectra and (b, c) elemental composition of interior and exterior char residues for pristine EP and EP/MnO2@CHS@SA@Ni 2% as determined by XPS

Table S1 TGA results of pristine EP, EP/MnO_2 and EP/MnO_2@CHS@SA@Ni composites under nitrogen atmosphere.

Samples	$T_{-5\%}$ (ºC)	T_{max} (ºC)	Residue (wt%)
EP	381	395	12.3
EP/MnO_2 2%	362	387	16.8
EP/MnO_2@CHS@SA@Ni 0.5%	359	383	15.6
EP/MnO_2@CHS@SA@Ni 1%	355	380	16.4
EP/MnO_2@CHS@SA@Ni 2%	348	375	17.7

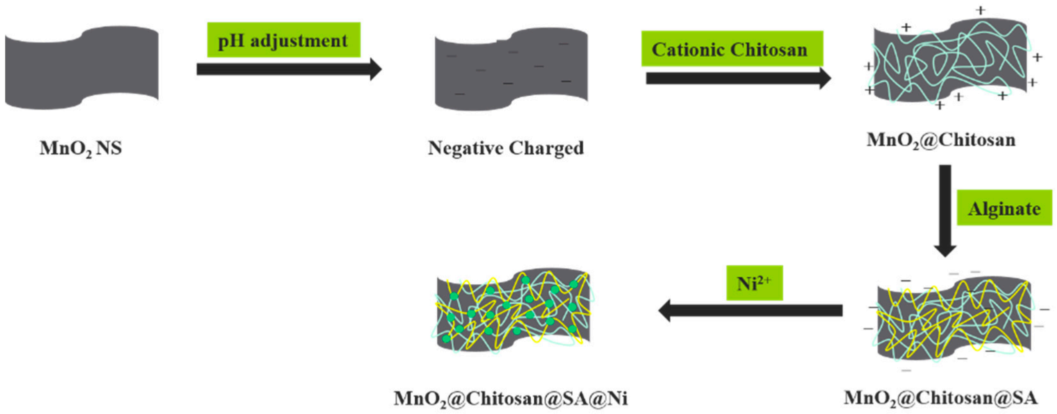

Figure S1 The synthesis process of MnO_2@CHS@SA@Ni hybrids.

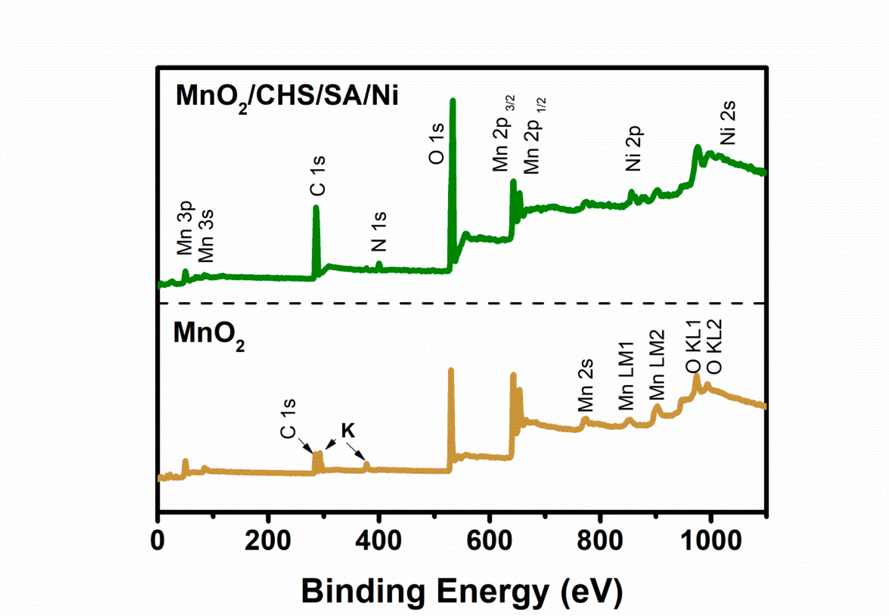

Figure S2 XPS survey spectra of MnO_2 and MnO_2@CHS@SA@Ni hybrids.

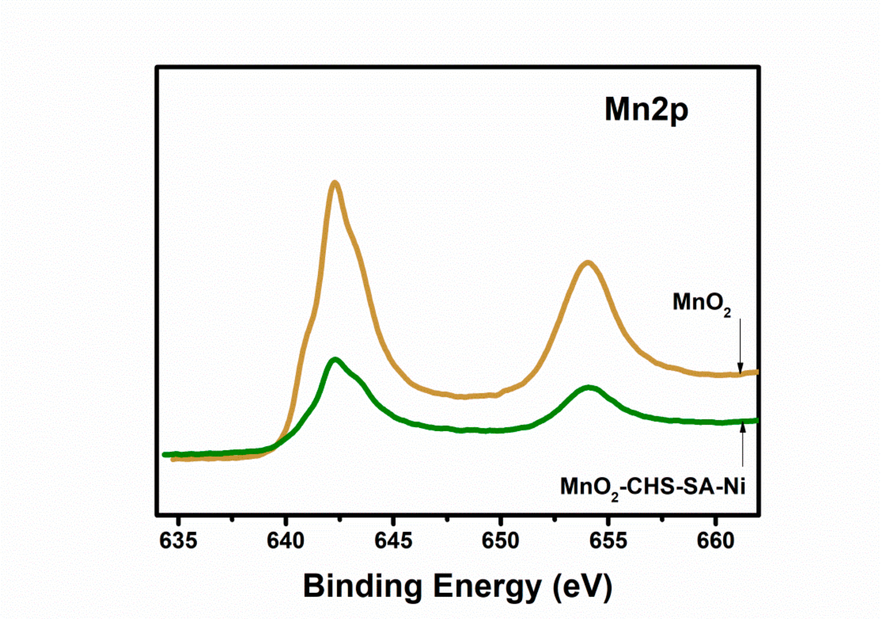

Figure S3 Mn 2p spectra of MnO_2 and MnO_2@CHS@SA@Ni hybrids.

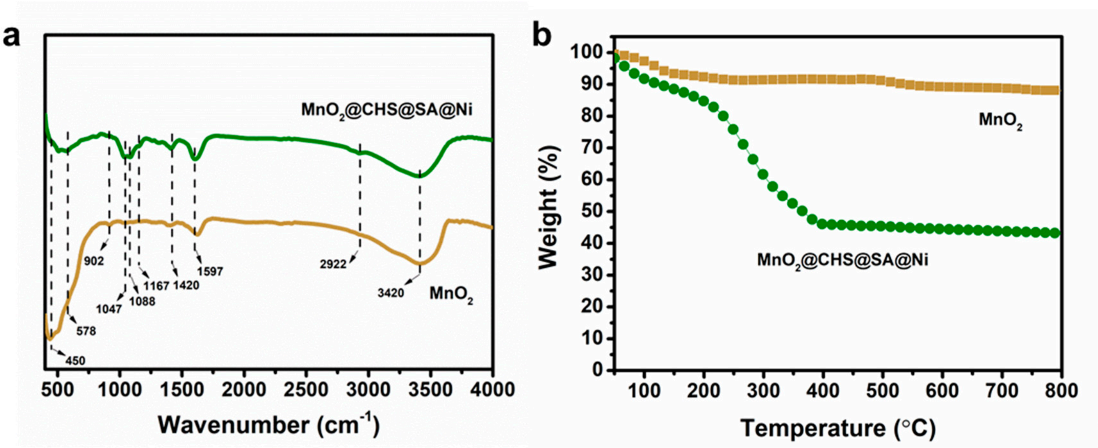

Figure S4 (a) FTIR spectra and (b) TGA curves of untreated MnO_2 and MnO_2@CHS@SA@Ni hybrids.

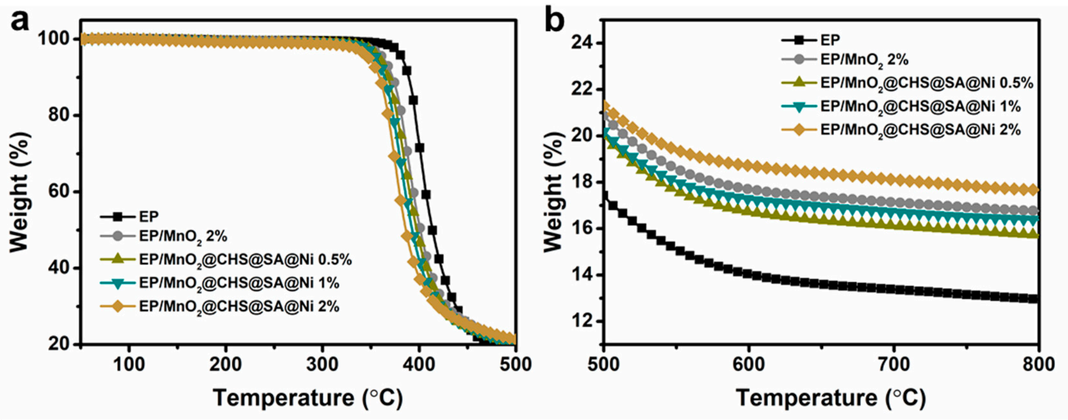

Figure S5 The enlarged TGA patterns **of pristine** EP, EP/MnO_2 and EP/ MnO_2@CHS@SA@Ni composites under nitrogen atmosphere.

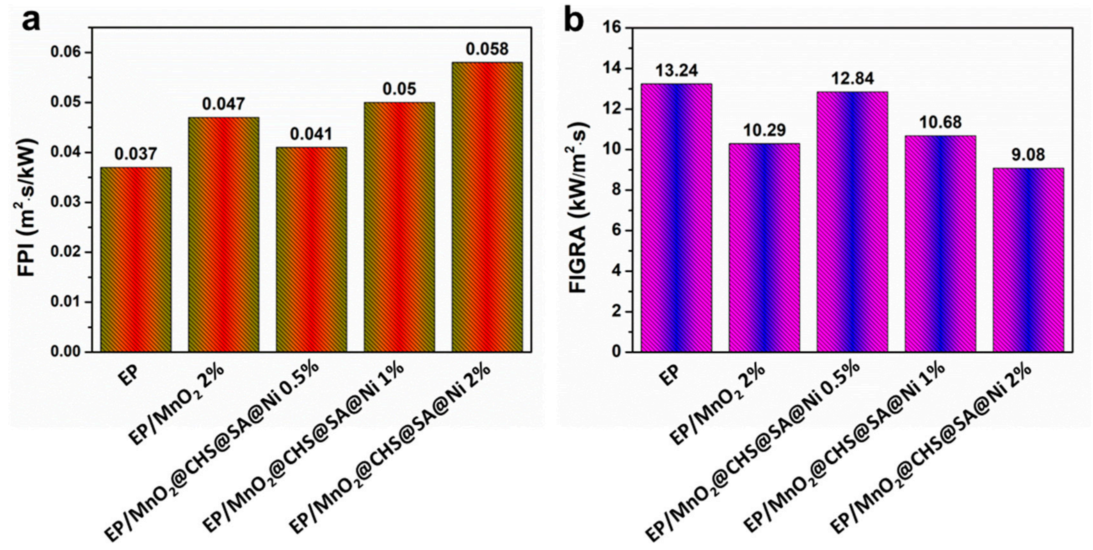

Figure S6 (a) FPI and (b) FIGRA values of **pristine** EP resin, EP/MnO_2 composites, and EP/MnO_2@CHS@SA@Ni composites.

a

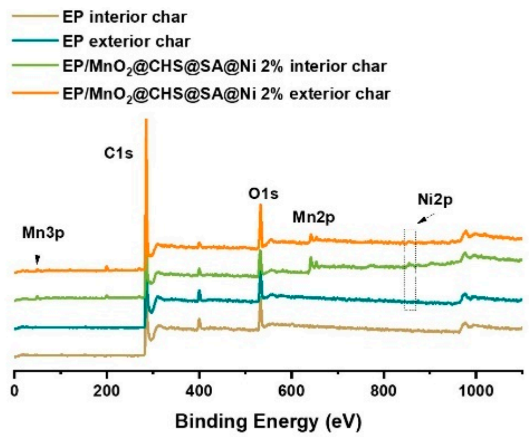

b Interior char

EP			$EP/MnO_2@CHS@SA@Ni$		
Name	Peak BE	Atomic %	Name	Peak BE	Atomic %
C1s	284.8	86.02	C1s	284.8	83.8
O1s	400.2	8.69	O1s	532.1	10.2
N1s	532.8	5.29	N1s	400.1	3.6
Mn2p	641.3	0	Mn2p	641.3	1.8
Ni2p	855.6	0	Ni2p	855.6	0.6

c Exterior char

EP			$EP/MnO_2@CHS@SA@Ni$		
Name	Peak BE	Atomic %	Name	Peak BE	Atomic %
C1s	284.8	86.73	C1s	284.8	82.3
O1s	400.2	8.7	O1s	532.1	12.9
N1s	532.8	4.57	N1s	400.1	3.4
Mn2p	641.3	0	Mn2p	641.3	1.0
Ni2p	855.6	0	Ni2p	855.6	0.4

Figure S7 Interior and exterior char residue analysis by using XPS technique. (a) XPS survey spectra and (b, c) elemental composition of interior and exterior char residues for pristine EP and $EP/MnO_2@CHS@SA@Ni$ 2% as determined by XPS.
